# Automated segmentation and diagnosis of pneumothorax on chest X-rays with fully convolutional multi-scale ScSE-DenseNet: a retrospective study

**DOI:** 10.1186/s12911-020-01325-5

**Published:** 2020-12-15

**Authors:** Qingfeng Wang, Qiyu Liu, Guoting Luo, Zhiqin Liu, Jun Huang, Yuwei Zhou, Ying Zhou, Weiyun Xu, Jie-Zhi Cheng

**Affiliations:** 1 School of Computer Science and Technology, Southwest University of Science and Technology, Mianyang, China; 2 Radiology Department, Mianyang Central Hospital, Mianyang, China; 3 Shanghai United Imaging Intelligence Co. Ltd., Shanghai, China

**Keywords:** Chest X-ray, Pneumothorax segmentation and diagnosis, fully convolutional DenseNet, Spatial and channel squeezes and excitation, Spatial weighted cross-entropy loss

## Abstract

**Background:**

Pneumothorax (PTX) may cause a life-threatening medical emergency with cardio-respiratory collapse that requires immediate intervention and rapid treatment. The screening and diagnosis of pneumothorax usually rely on chest radiographs. However, the pneumothoraces in chest X-rays may be very subtle with highly variable in shape and overlapped with the ribs or clavicles, which are often difficult to identify. Our objective was to create a large chest X-ray dataset for pneumothorax with pixel-level annotation and to train an automatic segmentation and diagnosis framework to assist radiologists to identify pneumothorax accurately and timely.

**Methods:**

In this study, an end-to-end deep learning framework is proposed for the segmentation and diagnosis of pneumothorax on chest X-rays, which incorporates a fully convolutional DenseNet (FC-DenseNet) with multi-scale module and spatial and channel squeezes and excitation (scSE) modules. To further improve the precision of boundary segmentation, we propose a spatial weighted cross-entropy loss function to penalize the target, background and contour pixels with different weights.

**Results:**

This retrospective study are conducted on a total of eligible 11,051 front-view chest X-ray images (5566 cases of PTX and 5485 cases of Non-PTX). The experimental results show that the proposed algorithm outperforms the five state-of-the-art segmentation algorithms in terms of mean pixel-wise accuracy (MPA) with $0.93\pm 0.13$ and dice similarity coefficient (DSC) with $0.92\pm 0.14$, and achieves competitive performance on diagnostic accuracy with 93.45% and $F_1$-score with 92.97%.

**Conclusion:**

This framework provides substantial improvements for the automatic segmentation and diagnosis of pneumothorax and is expected to become a clinical application tool to help radiologists to identify pneumothorax on chest X-rays.

## Background

Pneumothorax (PTX) is an acute pulmonary disease with respiratory disorder caused by the abnormal accumulation of air in the pleural space between the chest wall and the lung [1, 2]. According to the previous study in United States, PTX can occur in a variety of clinical settings and in individuals of any age, with a 35% recurrence rate in men [3]. PTX can cause pleuritic chest discomfort and dyspnea, and in severe cases may precipitate life-threatening medical emergency with cardio-respiratory collapse, requiring immediate intervention and subsequent prevention [4].

The screening and diagnosis of pneumothorax usually rely on chest radiographs that are formed by the differences in the absorption of X-ray ionizing radiation of different tissues in the chest [5]. Since chest radiographs project all three-dimensional anatomical clues of the chest onto a two-dimensional plane, the pneumothoraces in chest X-rays may be very subtle and overlapped with the ribs or clavicles. The identification of pneumothorax in chest X-ray is difficult and largely depends on the experience of radiologists. The failure of radiologists to detect PTX in early examination is one of the leading causes of PTX death [2]. Therefore, it is highly demanded to develop an automatic algorithm to reduce missed diagnosis and to help radiologists identify PTX accurately and timely.

Conventional PTX detection methods mainly consider the local and global texture cues [6], features from phase stretch transform (PST) [2], and local binary pattern (LBP) and then employ support vector machine (SVM) to classify the presence and absence of pneumothorax [7]. These conventional algorithms, which count on hand-crafted features and require prior knowledge for the feature engineering that can be well modeled through shape and appearance features and consistent data distribution, are suited to the detection of regular organs and lesions. However, the modeling capability of the conventional method is very limited when the shape and size of PTX vary greatly and the characteristics are not obvious.

Recently, deep learning-based technologies, especially the convolutional neural networks (CNNs), have shown great potential in medical image analysis [8, 9]. Several deep CNNs algorithms have been proposed for the identification of PTX with the image-level annotation. Wang et al. [10] released a large-scale chest X-ray dataset with image-level annotation, and proposed a deep CNN for the classification of 14 abnormalities (including PTX) on chest X-ray. This study is a milestone of PTX detection in the era of deep learning. Later, the studies of [11–14] proposed more accurate classification networks for the 14 kinds of chest diseases, and the studies of [4, 15] proposed methods that only detect PTX. Despite these deep learning-based methods have demonstrated effectiveness in the PTX identification with image-level annotation, the utilization of image-level annotation makes the localization of pneumothorax on chest X-ray insufficiently precise. Since the segmentation of PTX region can help determine the large PTX for the automatic triaging scheme [16], accurate segmentation of PTX with pixel-level annotation is very crucial to the accurate localization of pneumothorax. However, due to the difficulty in obtaining pixel-level annotations of PTX, there are few studies on PTX segmentation.

Lesion segmentation in medical images is the most fundamental tool for the support of lesion analysis and treatment planning. Automatic and accurate segmentation tool can better help radiologists in the quantitative image analysis and support precise diagnosis. In this study, we create a large chest X-ray dataset for pneumothorax with pixel-level annotation by radiologists and explore an automatic segmentation algorithm for PTX identification using fully convolutional networks (FCNs) [17]. FCNs were introduced in the literature as a natural extension of CNNs to formulate semantic segmentation as pixel classification problem. FCNs and its further extensions like U-Net [18] have achieved remarkable performance for several tasks like the segmentation of lungs, clavicles, heart in chest radiographs [19], brain tumors [20], estimation of cardiothoracic ratio [21], etc. However, the PTX areas in chest X-rays may be very subtle and varied in shape, overlapping with the ribs or clavicle, and therefore the PTX segmentation task suffers from pixel imbalance and multi-scale problems.

In this study, we propose a fully convolutional multi-scale scSE-DenseNet framework for PTX segmentation and diagnosis with the pixel-level annotation on chest X-ray. The framework consists of three modules: (1) a fully convolutional DenseNet (FC-DenseNet), which is parameter efficient and served as the backbone of the framework; (2) a multi-scale module that captures the variability of viewpoint-related objects and learns the relationships across image structures at multiple scales; (3) a scSE module, which is incorporated into each convolution layer in the dense block of FC-DenseNet and can adaptively recalibrate feature maps to elucidate useful features while suppressing non-useful features without adding much parameters. To tackle the imbalance problem of pixels [22], we also introduce a spatially weighted cross-entropy loss (SW-CEL) function to penalize the target areas, background and boundary pixels using different weights. The proposed method can not only reduce the impact of class imbalance, but also better describe the boundary areas to segment and diagnose pneumothorax accurately. This study extends our preliminary work [23] by redesigning the automatic segmentation and diagnosis framework for PTX, adding extensive experiments to evaluate the automatic segmentation and diagnosis of PTX, and discussing the effects of different growth rates and loss functions on PTX segmentation.

## Methods

**Fig. 1:**
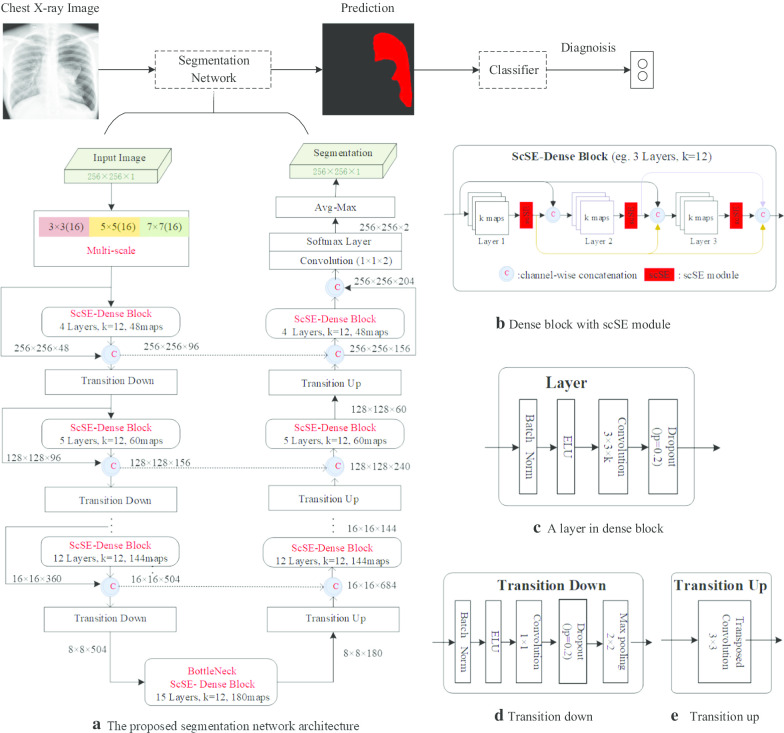
The automatic segmentation and diagnosis framework for pneumothorax on chest X-rays. **a** The proposed segmentation network architecture. The difference between our segmentation network and the original FC-DenseNet is marked in red on the subgraph. **b** An example of a dense block embedded with scSE modules. **c** A layer in the scSE-embedded dense block that consists of batch normalization, exponential linear unit, $3\times 3$ convolution operation, and drop-out rate $\rho =0.2$. **d** A transition down block, which is composed of batch normalization, exponential linear unit, $1\times 1$ convolution, dropout ($\rho =0.2$) and $2\times 2$ max pooling. (e) A transition up block, which is composed of $3\times 3$ transposed convolution

In this section, an end-to-end deep learning framework is proposed for PTX segmentation by using FC-DenseNet as a backbone with the embedding of multi-scale module and scSE module, and a simple classifier is added to set the threshold to diagnose PTX by classifying the predicted PTX segmentation maps, as shown in Fig. 1.

### Fully convolutional DenseNet for PTX segmentation

A deep learning-based typical segmentation architecture is composed of two parts: a down-sampling path (contraction) and an up-sampling path (expansion), where the down-sampling path is responsible for feature learning and the up-sampling path aims to restore the spatial information and image resolution. Alternatively, skip connections can be used to help the up-sampling path to recover spatial detail information from the down-sampling path by reusing feature maps. In this study, we employ FC-DenseNet [24, 25] as the network backbone for its advantages of parameters reduction, computational efficiency and better withstand of over-fitting problem.

The down-sampling path of FC-densenet consists multiple blocks, each containing a dense block followed by a transition-down block. For each dense block, it iteratively concatenates all feature maps in a feedforward paradigm. A dense block contains multiple layers, each consisting of a batch normalization, a non-linearity activation function, a convolution operation, and a dropout connection (see Fig. 1c). Each layer in the dense block, *l*, takes all feature maps of the preceding layers that match the spatial resolution as input, outputs *k* feature maps and passes them to the subsequent layers (see Fig. 1b), where *k* is known as growth rate. Hence, the number of feature maps in the dense block grows linearly with the depth of the down-sampling path of FC-DenseNet and the output of the $l{\mathrm{th}}$ layer can be defined as:

1 $$\begin{aligned} x_l = H_l (x_{l-1}\oplus x_{l-2}\oplus \cdots \oplus x_0). \end{aligned}$$

where $x_l$ denotes the feature maps at the $l{\mathrm{th}}$ layer, the notation $\oplus$ denotes the channel-wised concatenation for the feature maps from the layer $l-1$ to the layer 0. *H* is a composition of batch normalization, exponential linear unit and convolutional layer with dropout rate of 0.2 (see Fig. 1c), and $H_l$ represents a composite function of the $l{\mathrm{th}}$ layer.

In order to reduce the spatial dimensionality of the feature maps, a transition down block following the dense block is introduced (see Fig. 1d). The transition down block consists of batch normalization, exponential linear unit and $1\times 1$ convolution for depth preserving with dropout rate of 0.2, and followed by a $2\times 2$ max pooling operation. In particular, the end block of the down-sampling path is called bottleneck and is connected to the up-sampling path.

Through the up-sampling path, the spatial resolution of the input can be recovered by transition up blocks, dense blocks, and skip connections from the corresponding blocks of the down-sampling path. The transition up block is a transposed $3\times 3$ convolution (see Fig. 1e), which implements the up-sampling of the previous feature maps. Then, the up-sampled feature maps are channel-wisely concatenated with the feature maps from the corresponding skip connections in the down-sampling path as the input of the dense block in the up-sampling path. At the end of up-sampling path, the feature maps of the output are convolved with a $1\times 1$ convolution layer, and followed by a softmax layer and average max-pooling operation to generate the final segmentation map. This connection pattern strongly encourages the reuse of features and allows all layers of the architecture to receive direct supervision signals.

### Multi-scale convolution module

To learn the relations across lesion features on multiple scales, multiple convolution kernels with different receptive fields were parallelly incorporated into the first convolution layer of FC-DenseNet to capture variability of viewpoint-related object. The module for processing chest X-ray images with varying size of convolution kernels is called the multi-scale convolution module. GoogLeNet [26] has introduced the multi-scale convolution kernels into a parallel sub-network as a inception module, allowing the abstract convolution features with different scales to be transported to the subsequent layer simultaneously. The inception module of GoogLeNet contains different size of convolution filters such as $1\times 1$, $3\times 3$ and $5\times 5$ convolutional kernels, and $3\times 3$ max-pooling operation.

**Fig. 2:**
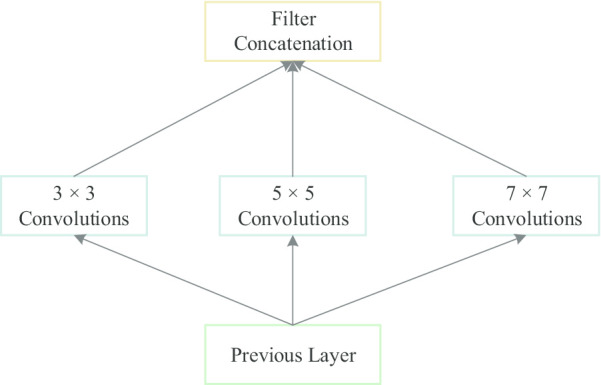
Multi-scale convolution module. A modified version of inception module by removing $1\times 1$ convolution kernel and $3\times 3$ max-pooling and adding a larger convolution kernel ($7\times 7$) to expand the receptive field

In the semantic segmentation task, a small convolution kernel can help the detection of small target regions, and a larger convolution kernel can not only detect the larger target regions, but also eliminate the false positive regions. Therefore, we add a larger convolution kernel ($7\times 7$) to expand the receptive field for the segmentation of PTX. To avoid the reduction of segmentation accuracy caused by dimension reduction, we also removed the $1\times 1$ convolution kernel and $3\times 3$ max-pooling, making the multi-scale convolution kernel module more efficiently in the PTX segmentation architecture. After these different convolution operations, all feature maps are channel-wisely concatenated for the subsequent dense block (see Fig. 2)

### Spatial and channel squeezes and excitation (scSE) module

Most of fully convolutional networks (FCNs)-based segmentation methods mainly focus on the joint space and channel encoding. For example, FC-DenseNet can simultaneously transmit the spatial and channel information of the current filters to the subsequent convolution layers to improve the utilization of features. However, spatial- and channel-wise independent coding are less utilized. Recently, Hu et al. [27] proposed a framework embedded with squeeze and excitation (SE) blocks to model the interdependencies between feature channels, and achieved state-of-the-art results in image classification. Roy et al. [28] introduced three variants of the SE blocks, including the channel SE (cSE) module, the spatial SE (sSE) module, and the concurrent spatial and channel squeeze and excitation (scSE) module, to migrated the SE blocks from image classification to image segmentation with promising performance. The purposes of the SE and cSE module are to adaptively recalibrate feature maps along the channels and to elucidate useful channels while suppressing the less useful channels. The cSE module can only reweight channels and the sSE module can only reweight spaces, while the scSE module can recalibrate the feature maps of channels and spaces respectively, and then merge these feature maps into output layer.

**Fig. 3:**
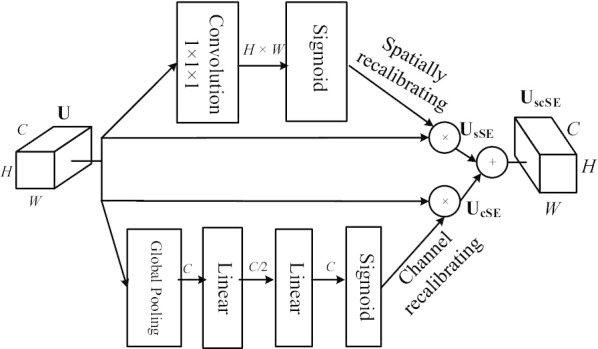
The concurrent spatial and channel squeeze and excitation (scSE) module. The input feature maps of a dense block ${\mathrm {U}}$ can be recalibrated to the output feature maps ${{\mathrm {U}}}_{{\mathrm{scSE}}}$ through the two branches of ${{\mathrm {U}}}_{{\mathrm{sSE}}}$ and ${{\mathrm {U}}}_{{\mathrm{cSE}}}$. The top branch is the spatial recalibrating (${\mathrm {U}}_{{\mathrm {sSE}}}$), and the bottom branch is channel-wise recalibrating (${\mathrm {U}}_{{\mathrm {cSE}}}$), and then ${\mathrm {U}}_{{\mathrm {sSE}}}$ and ${\mathrm {U}}_{{\mathrm {cSE}}}$ are merged into the output

In this study, we embedded the scSE module into each dense block and proposed the application of scSE dense block in pneumothorax segmentation (see Fig. 1a, b). We denote the input feature maps of a dense block as ${\mathrm {U}}$, ${\mathrm {U}}\in {\mathbb {R}}^{H\times W\times C}$, where *H*, *W*, and *C* denote the spatial height, width, and the number of channels, respectively. As illustrated in Fig. 3, the input feature maps ${\mathrm {U}}$ can be recalibrated to the output feature maps ${{\mathrm {U}}}_{{\mathrm{scSE}}}$, ${{\mathrm {U}}}_{{\mathrm{scSE}}}\in {\mathbb {R}}^{H\times W\times C}$, through the two branches of ${{\mathrm {U}}}_{{\mathrm{sSE}}}$ and ${{\mathrm {U}}}_{{\mathrm{cSE}}}$. The ${{\mathrm {U}}}_{{\mathrm{scSE}}}$ can be formulated as:

2 $$\begin{aligned} {{\mathrm {U}}}_{{\mathrm{scSE}}}={{\mathrm {U}}}_{{\mathrm{sSE}}} + {{\mathrm {U}}}_{{\mathrm{cSE}}} \end{aligned}$$

where ${{\mathrm {U}}}_{{\mathrm{sSE}}}$ and ${{\mathrm {U}}}_{{\mathrm{cSE}}}$ are recalibrated from ${\mathrm {U}}$ in spatial space and on the channels, respectively. ${{\mathrm {U}}}_{{\mathrm{sSE}}}$ can provide more relevant spatial locations by ignoring irrelevant spatial locations and ${{\mathrm {U}}}_{{\mathrm{cSE}}}$ can be adaptively tuned to ignore less important channels and to emphasize more important channels.

**Fig. 4:**
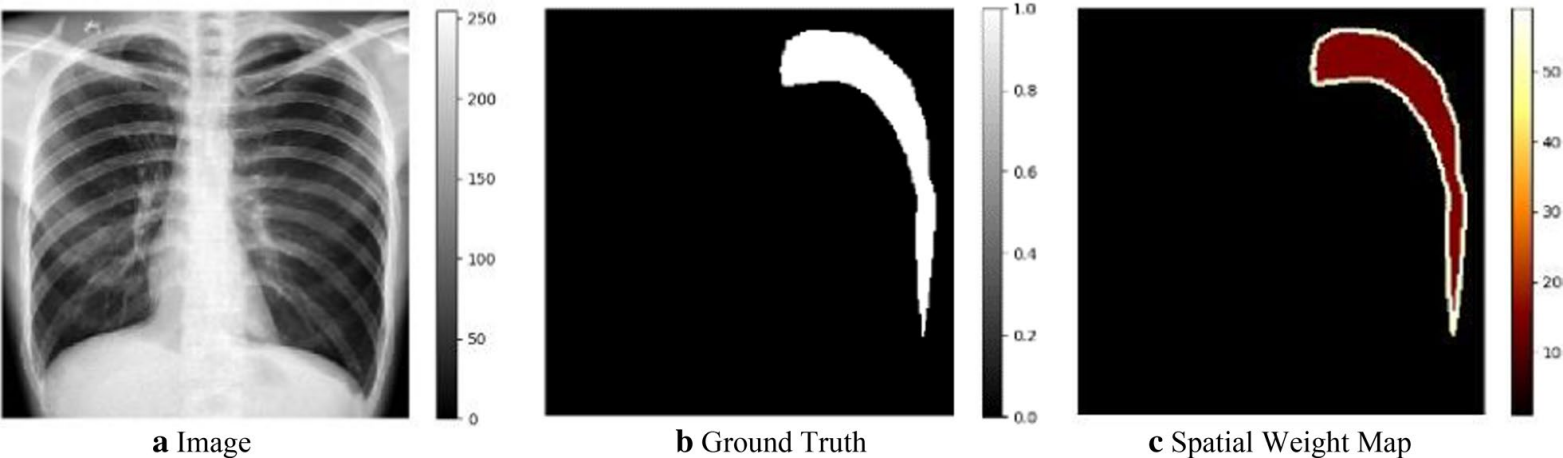
The process of generating the spatial weight map. The groud-truth image **b** is delineated by radiologist according to the chest X-ray image **a**. Through edge detection and morphological dilation of the boundary contour pixels of the target class, the spatial weight map **c** can be generated from the ground-truth image **b**. The colors in the spatial weight map represent the weight distribution according to its relative class frequency

Specifically, ${{\mathrm {U}}}_{{\mathrm{sSE}}}$ can be obtained from ${\mathrm {U}}$ through a $1\times 1\times 1$ convolution kernel and a sigmoid function. The computing weight of the convolution kerner, denoted as $W_s$, $W_s\in {\mathbb {R}}^{1\times 1\times C\times 1}$, can be used to learn a projection tensor *Q*, where $Q\in {\mathbb {R}}^{H\times W}$. Then the sigmoid function $\sigma (\cdot )$ is applied to rescale the activations of *Q* into [0, 1]. Hence, ${{\mathrm {U}}}_{{\mathrm{sSE}}}$ can be defined as:

3 $$\begin{aligned} {{\mathrm {U}}}_{{\mathrm{sSE}}}=\sigma (W_s \times {\mathrm {U}}) \end{aligned}$$

For the cSE module, a global average pooling operation $g(\cdot )$ is first performed on the input feature maps ${\mathrm {U}}$ to generate a vector *z* embedded with globally spatial information, where $z=g({\mathrm {U}})$, $z\in {\mathbb {R}}^{1\times 1\times C}$. Then two consecutive fully connected layers are used to convert the vector *z* into a new vector $\hat{z}$, $\hat{z}=W_1(\delta (W_2 z))$, where $W_1$ ($W_1\in {\mathbb {R}}^{C\times \frac{C}{2}}$) and $W_2$ ($W_2\in {\mathbb {R}}^{\frac{C}{2}\times C}$) denote the weights of the two consecutive fully connected layers, respectively, and $\delta (\cdot )$ denotes the operation of ReLU. Afterwards, we apply a sigmoid function $\sigma (\cdot )$ to normalize the activations into [0, 1]. Therefore, the formulation of cSE module can be defined as:

4 $$\begin{aligned} {{\mathrm {U}}}_{{\mathrm{cSE}}}=\sigma (W_1(\delta (W_2(g({\mathrm {U}})))) \end{aligned}$$

In summary, the scSE module combines the advantages of sSE module and cSE module, enabling better adaptive recalibration of feature maps, so that the scSE dense block can elucidate more useful information while suppressing less useful features in the application of pneumothorax segmentation.

### Spatially weighted cross-entropy loss

The serious pixel class imbalance issue between the region of interests (ROIs) and the surrounding background generally exists in medical image segmentation. The number of pixels with pathology is much less than that without pathology. This tends to cause the learning model to fall into a local minimum. The typical cross entropy loss (CEL), which measures the quantization error of all pixels by calculating the pixel-level probabilistic error between the predicted output class and the target class, is susceptible to the class imbalance problem. Then weighted cross-entropy loss (W-CEL) is introduced to mitigate the effect of class imbalance by giving different weights to target classes and background pixels. Meanwhile, dice loss [29] is also proposed to optimize the dice overlap coefficient between the predictive segmentation map and the ground truth map. However, due to the narrow boundary of the pneumothorax class, it is still difficult to distinguish the target classes from the background pixels through W-CEL and dice loss. Therefore, the boundary class is also required to be considered along with the target and background classes.

Pneumothorax segmentation is generally formulated as a binary classification task with respect to object (pneumothorax) versus background, where ‘0’ is used to represent the background pixels and ‘1’ is used to represent the pneumothorax pixels. In this study, if the eight neighborhoods of the pixel value ‘1’ have a pixel value of ‘0’, we define this pixel value ‘1’ as boundary contour pixels. To formulate the boundary contour pixels of pneumothrax, an edge detector is used to determine whether a pixel is a boundary pixel or not, and then the boundary range is cross-expanded by morphological dilation. Therefore, a spatial weighted cross-entropy loss (SW-CEL) is proposed by considering the different weights of target, background and boundary [30]. As shown in Fig. 4, spatial weight maps generated from the ground-truth images are used to calculate the weight loss of each pixel in the cross-entropy loss. The spatially weighted cross-entropy (SW-CEL) loss can be formulated as:

5 $$\begin{aligned} {\mathcal {L}}(X;W)=-\sum _{x_i\in X}w_{map}(x_i)log (p(t_i|x_i;W)) \end{aligned}$$

where *X* denotes the training samples, *W* denotes the set of learnable weights, $W=(w_1, w_2, \ldots , w_l)$, and $w_l$ denotes the weight matrix of the $l{\mathrm{th}}$ layer. $p(t_i|x_i;W)$ represents the probability prediction for a pixel $x_i$, and $t_i$ is the target label of the pixel $x_i$, $(x_i\in X)$. $w_{map}(x_i)$ is the estimated weight for each pixel $x_i$, which can be defined as:

6 $$\begin{aligned} w_{map}(x_i)=\sum _{c\in C} \frac{|N|}{|T_c|}*F_T(x_i)+\frac{|N|}{|B_c|}*F_B(x_i) \end{aligned}$$

where $$F_T(x_i)=\left\{ \begin{array}{ll} 0, &{}x_i\notin T_c \\ 1,&{} x_i\in T_c \end{array} \right.$$ and $$F_B(x_i)=\left\{ \begin{array}{ll} 0, &{}x_i\notin B_c \\ 1, &{}x_i\in B_c \end{array} \right.$$. *C* denotes the set of all ground truth classes, i.e., pneumothorax class and background class. For each chest X-ray image, *N* denotes the set of total pixels and $T_c$ denotes the set of pixels corresponding to each class c, $c\in C$, and $B_c$ denotes the boundary contour pixel set, $B_c \subset T_c \subset N$. $F_T(x_i)$ and $F_B(x_i)$ denote the indicator functions defined on the subsets $T_c$ and $B_c$, respectively.

### Automated classification for pneumothorax diagnosis

Most of previous studies of pneumothorax on chest X-ray mainly focous on PTX or not PTX diagnosis with image-level annotation. The learning of pneumothorax diagnosis with image-level annotation is a typical weakly supervised learning method, which often leads to inaccurate locations of pneumothorax lesions because the locations of pneumothorax lesions are not marked. Pneumothorax segmentation can accurately provide pixel-level lesion locations and better assist radiologists in pneumothorax diagnosis. In this study, we propose a pixel-wise level supervised network for the automatic segmentation and diagnosis of PTX (see Fig. 1). Since the predicted segmentation maps are the result of binary pixel-wise classification network, a simple classifier is added and a threshold is set to classify the predicted segmentation maps. We specify that if the predicted segmentation map is greater than a threshold, it is pneumothorax; otherwise, it is non-pneumothorax. If the threshold is too small, it may be segmentation noise; If the threshold is too high, small pneumothorax may be missed. Therefore, the threshold is empirically set to 50 pixels for pneumothorax diagnosis according to the predicted segmentation maps.

### Dataset

The study data was conducted with three-stage procedures. The first stage searched a keyword “pneumothorax” in picture archiving and communications system (PACS) of our institution to obtain all relevant chest radiographs and radiology reports. In second stage, the key word “pneumothorax” was identified in the radiological report, and those without pneumothorax were classified as non-pneumothorax (Non-PTX) group, while those with pneumothorax were classified as pneumothorax (PTX) group. Third, all image data in PTX group was pixel-wisely annotated by three medical students and then revised by an experienced radiologist.

**Table 1:** Data distribution description of each subset

Subset	PTX	Non-PTX	Total	Percentage (%)
Training	3617	3453	7070	64
Validation	866	902	1768	16
Test	1083	1130	2213	20
Total	5566	5485	11051	100

Our eligible sample included a total of 11,051 front-view chest X-ray images (5566 cases of PTX and 5485 cases of Non-PTX). We named this dataset as “PX-ray”. As shown in Table 1, the PX-ray dataset was randomly divided into the training, validation and test sets by stratified sampling strategy, so as to ensure that the ratio of PTX group and Non-PTX group in each set was the same.

### Evaluation

To evaluate the performance of the PTX segmentation network, we used three quantitative metrics: mean pixel-wise accuracy (MPA), dice similarity coefficients (DSC) and Hausdorff distance (HD). Statistical tests were also used to show whether there are significant differences in the results of different segmentation algorithms. If the *p* value of the statistical test is less than 0.05, there is a significant difference between the two results.

MPA is the average ratio of the accuractely classified pixels on the classes of PTX and non-PTX, defined as:

7 $$\begin{aligned} {\mathrm {MPA}} = \frac{1}{N*C}\sum _{n=1}^{N}\sum _{c=1}^{C}\frac{p_c}{P_c} \end{aligned}$$

where *N* denotes the number of samples, *C* denotes the number of classes, $p_c$ denotes the number of the accuractely classified pixels of class *c*, and $P_c$ denotes all pixels of class *c* in the ground truths. More importantly, we defined the pixel-wise accuracy (PA) of the PTX group class as ${\mathrm {PA}}_1$.

8 $$\begin{aligned} {\mathrm {PA}}_1 = \frac{1}{N}\sum _{n=1}^{N}\frac{p_1}{P_1} \end{aligned}$$

DSC is a standard measure for segmentation evaluation by calculating the overlap rate between the ground-truth map and the predicted segmentation map.

9 $$\begin{aligned} {\mathrm {DSC}} = \frac{1}{N}\sum _{c=1}^{C}\frac{2(A_c\cap B_c)}{A_c+B_c} \end{aligned}$$

where $A_c$ denotes all pixels of class *c* in the predicted segmentation map and $B_c$ denotes all pixels of class *c* in the ground-truth map. More importantly, we define the DSC of the PTX group class as ${\mathrm {DSC}}_1$.

10 $$\begin{aligned} {\mathrm {DSC}}_1 = \frac{1}{N}*\frac{2(A_1\cap B_1)}{A_1+B_1} \end{aligned}$$

Hausdorff (HD) metric is also used to measure the contour distance between the ground-truth map and the predicted segmentation map, which can be defined as:

11 $$\begin{aligned} H(P, G)= & {} \max (h(P,G),h(G,P)) \end{aligned}$$

12 $$\begin{aligned} h(P,G)= & {} \max _{p_i\in P}\min _{g_i\in G}||p_i-g_i|| \end{aligned}$$

where *P* and *G* are the pixel sets of the predicted segmentation map and the ground-truth contours, respectively. The smaller the Hausdorff value, the higher the matching degree of the two contours.

### Detailed settings

All experiments in this study were conducted on Nvidia Tesla V100 GPU server. The weights of the PTX segmentation network were initialized with HeUniform [31]. We used Adam optimizer ($\beta _1=0.9$, $\beta _2=0.999$) with learning rate of 1e${-}$4 and weight decay of 1e${-}$4 to train the segmentaion network model for 200 epochs. During the training process of all models, data augmentation was performed by random horizontal flips and the validation set was used to early stop the training process. We monitored the dice similarity coefficient (DSC) score in the pneumothorax group with patience value of 20 epochs.

**Table 2:** Result comparisons of different segmentation models

Method	MPA	${\mathrm {PA}}_1$	DSC	${\mathrm {DSC}}_1$	Parameters	GFLOPS
U-Net [18]	0.90(0.16)*	0.81(0.31)*	0.89(0.17)*	0.79(0.33)*	7,764,098	*11.59*
SegNet [32]	0.91(0.15)*	0.81(0.31)*	0.90(0.16)*	0.80(0.31)*	29,444,162	40.14
DeepLab v3+ [33]	0.90(0.15)*	0.81(0.30)*	0.89(0.16)*	0.78(0.32)*	59,351,458	61.07
DenseASPP [34]	0.90(0.15)*	0.81(0.30)*	0.89(0.16)*	0.78(0.32)*	35,365,762	39.11
FC-DenseNet [25]	0.91(0.15)*	0.83(0.29)*	0.91(0.15)	0.82(0.29)	*5,415,278*	15.39
MS_scSE_U-Net	0.92(0.14)	0.85(0.28)	0.90(0.15)*	0.81(0.31)*	8,204,011	11.6
*MS_scSE_FC-DenseNet (Ours)*	*0.93(0.13)*	*0.86(0.27)*	*0.92(0.14)*	*0.84(0.27)*	5,989,096	15.59

The number with * represents a significant difference comparing other methods to our method, according to student’s T-test for two independent samples ($p<0.05$)

**Fig. 5:**
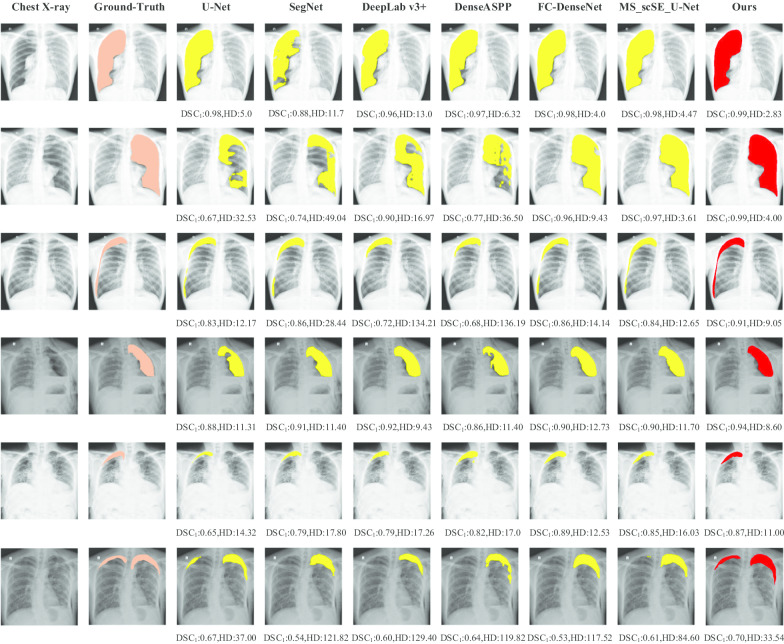
Segmentation result cases of large, moderate and small pneumothorax with U-Net, SegNet, Deeplab v3+, DenseASPP, FC-DenseNet, MS_scSE_U-Net and our proposed method MS_scSE_FC-DenseNet, as well as the corresponding DSC$_{\mathbf{1}}$ scores and HD scores. The segmentation results of ground truth, comparison methods and our proposed method are marked pastel orange, yellow and red, respectively

## Results

The qualitative and quantitative evaluation experiments are carried out to show the effectiveness of our proposed PTX segmentation and diagnosis framework. We first compare the performance of our network with that of U-Net [18], SegNet [32], DeepLab v3+ [33], DenseASPP [34] and original FC-DenseNet [25]. To verify the efficacy of the embedded modules in our segmentation and diagnosis network, we also embed the multi-scale module and scSE modules into U-Net and develop a new architecture, named as “MS_scSE_U-Net”, for comparison. Note that the above segmentation and diagnosis networks share the same hyper-parameters and loss function SW-CEL during training.

**Table 3:** The quantitative evaluation of pneumothorax diagnosis results with different models

Models	Accuracy (%)	Sensitivity (%)	Specificity (%)	PPV (%)	NPV (%)	$F_1$-score (%)
U-Net [18]	88.39	81.16	95.31	94.31	84.07	87.24
SegNet [32]	90.78	85.87	95.49	94.80	87.58	90.11
DeepLab v3+ [33]	91.10	85.60	96.37	95.76	87.47	90.40
DenseASPP [34]	91.46	86.33	96.37	95.80	88.04	90.82
FC-DenseNet [25]	92.18	84.76	*99.29*	*99.14*	87.18	91.39
MS_scSE_U-Net	90.96	85.96	95.75	95.10	87.68	90.30
*MS_scSE_FC-DenseNet (Ours)*	*93.45*	*88.55*	98.14	97.86	*89.94*	*92.97*

### Performance of PTX segmentation

Table 2 shows that our PTX segmentation network, i.e., MS_scSE_FC-DenseNet, outperforms U-Net, SegNet, DeepLab v3+, DenseASPP and original FC-DenseNet in terms of MPA with $0.93\pm 0.13$, ${\mathrm {PA}}_1$ with $0.86\pm 0.27$, DSC with $0.92\pm 0.14$ and ${\mathrm {DSC}}_1$ with $0.84\pm 0.27$. Meanwhile, our network MS_scSE_FC-DenseNet performs better than the original FC-DenseNet, and MS_scSE_U-Net performs better than the original U-Net, which shows that the performance of the network embedded with the multi-scale module and scSE modules is better than that without them. This indicates that the proposed multi-scale module and scSE module play an important role in improving the performance of the segmentation networks for PTX. In addition, compared with the original FC-DenseNet, the parameter number of the proposed network increased by 10.59%, but is still much less than that of other segmentation networks. Our method has a low time cost in terms of giga floating-point operations per second (GFLOPS).

**Table 4:** Evaluation of pneumothorax segmentation performance with different growth rate *k*

Growth rate mean (standard deviation)								
PA	$k=2$	$k=4$	$k=6$	$k=8$	$k=10$	$k=12$	$k=14$	$k=16$
${\mathrm {PA}}_1$	0.81(0.32)*	0.84(0.28)*	0.84(0.28)*	0.85(0.27)	0.858(0.26)	*0.859(0.27)*	0.857(0.26)	0.85(0.27)
MPA	0.90(0.16)*	0.92(0.14)*	0.92(0.14)*	0.92(0.14)	0.928(0.13)	*0.928(0.13)*	0.927(0.13)	0.92(0.13)

DSC	$k=2$	$k=4$	$k=6$	$k=8$	$k=10$	$k=12$	$k=14$	$k=16$
DSC1	0.80(0.32)*	0.81(0.30)*	0.82(0.29)*	0.82(0.29)*	0.832(0.27)	*0.838(0.27)*	0.833(0.28)	0.83(0.30)
DSC	0.90(0.16)*	0.91(0.15)*	0.91(0.15)*	0.91(0.14)*	0.915(0.14)	*0.918(0.14)*	0.915 (0.14)	0.91(0.14)

The values are provided in the form of mean (standard deviation)

Figure 5 shows some result cases of large, moderate and small pneumothorax with different segmentation algorithms. For each case, we present the orginal chest X-ray image, the ground-truth image, and the segmentation results of the comparison methods and our proposed method MS_scSE_FC-DenseNet, as well as the corresponding ${\mathrm {DSC}}_1$ and HD scores. It can be found that our method performs better with a larger ${\mathrm {DSC}}_1$ and a smaller HD scores, which can more accurately help radiologists find the pneumothorax area. In addition, as shown in the bottom line of Fig. 5, our algorithm can segement the small bilateral thoracic regions that are very difficult for radiologists to manually label, indicating the potential of our method for clinical computer-assisted diagnosis.

**Fig. 6:**
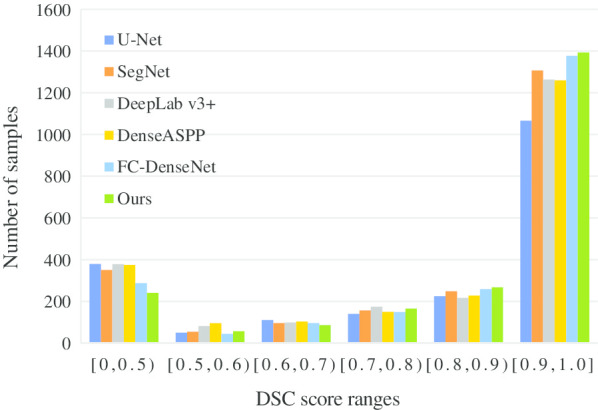
Qualitative evaluation of our proposed PTX segmentation network againsts with the five comparison frameworks for the PTX segmentation task. The Y-axis represents the number of contributed samples in the test dataset, and the X-axis represents the intervals of DSC for each model (columns)

Figure 6 shows qualitative evaluation of our proposed PTX segmentation network and the five comparison frameworks on the PTX segmentation task. The X-axis represents the intervals of DSC score, and the Y-axis represents the number of samples falling into the DSC intervals from the X-axis. Compared with other frameworks, our segmentation network has the largest number of sample size in the range of [0.9, 1.0] and the smallest sample size in the range of [0, 0.6].

**Table 5:** Evaluation of segmentation results with different loss function

Method	U-Net [18]	SegNet [32]	DeepLab v3+ [33]	DenseASPP [34]	FC-DenseNet [25]	MS_scSE_FC-DenseNet
${\mathrm {DSC}}_1$ Mean(Standard Deviation)						
CEL	0.78(0.34)	*0.81(0.31)*	0.78(0.33)	0.77(0.33)	0.82(0.30)	0.82(0.29)*
W-CEL	0.79(0.34)	0.80(0.32)	0.77(0.34)	0.76(0.34)*	*0.83(0.29)*	0.81(0.30)*
*SW-CEL*	*0.79(0.33)*	0.80(0.31)	*0.78(0.32)*	*0.78(0.32)*	0.82(0.29)	*0.84(0.27)*
HD Max(Mean)						
CEL	19.63(3.24)*	17.21(2.53)	*17.90(2.80)*	20.48(3.20)*	15.47(2.23)	17.81(2.27)*
W-CEL	19.99(2.80)*	17.21(2.53)	18.41(3.01)	19.74(3.71)	16.22(2.03)*	15.25(2.03)
*SW-CEL*	*17.76(2.60)*	*16.34(2.27)*	19.56(2.99)	*18.52(2.74)*	*14.81(2.02)*	*14.87(1.95)*

### Performance of pneumothorax diagnosis

The quantitative performances of pneumothorax diagnosis with different models are shown in Table 3. Our network shows the best results in terms of accuracy, sensitivity, negative predictive value (NPV) and $F_1$-score. The original FC-DenseNet shows the best performance on specificity and positive predictive value (PPV). In addition, all the segmentation networks used for PTX diagnosis achieve good performance. This indicates great potential for the pixel-wise level supervised networks. The pixel-level supervised network not only provides image-level information but also provides pneumothorax location and size information, which is of great help to network learning.

## Discussion

In this section, we discuss the effects of different gowth rates and loss functions on the pneumothorax segmentation performance.

### The effect of different growth rates

Table 4 discusses that our pneumothorax segmentation network performance with different growth rate (*k*) parameters. Note that according to students’ t-test of the two independent samples, the number with $*$ in the table represents that there is a significant difference ($p<0.05$) between the model with $k=12$ and other models. We can see that under the same framework, the results grow steadily as the value of *k* increases. The segmentation network with $k=12$ shows the best performance. Therefore, we use $k=12$ model as our final network for pneumothorax segmentation.

### The effect of different loss functions

Table 5 discusses the segmentation performance of three different loss functions, including CEL, W-CEL and SW-CEL. In order to further evaluate the performance of the loss function, we carry out experiments on our proposed network and the previous state-of-the-art networks including U-Net [18], SegNet [32], DeepLab v3+ [33], DenseASPP [34] and FC-DenseNet [25]. The statistical T-tests on the test set indicates that models trained on SW-CEL had no statistical significance in terms of DSC scores, while most models trained with SW-CEL showed the best performance in terms of Hausdorff distance scores. This indicates that the weight penalty for contour pixels could help to learn boundary contour accurately.

## Conclusion

In this study, we proposed a fully convolutional multi-scale scSE-DenseNet framework for automatic pneumothorax segmentation and diagnosis, which incorporates the advantages of feature reuse of DenseNet and greatly reduces a large number of parameters. We used the multi-scale module to capture the variability of viewpoint-related objects, as well as the scSE modules to conduct adaptive recalibration of the feature map and to boost meaningful features for better performance. To tackle the imbalance problem of pixels, SW-CEL was also introduced to better extract the pneumothorax boundaries on chest X-rays. The experiments conducted on PX-ray dataset demonstrate that our proposed framework is superior to the five state-of-the-art segmentation architectures in terms of MPA and DSC scores. This framework provides substantial improvements for the automatic segmentation and diagnosis of pneumothorax and is expected to become a clinical application tool for the pneumothorax segmentation and diagnosis.

## Data Availability

The dataset are available from the link: https://pan.baidu.com/s/1A47rQZ2H9IYVGC0jDS-j7Q, with the extract code: caev.

## Abbreviations

PTX: Pneumothorax
Non-PTX: Non-pneumothorax
FC-DenseNet: Fully convolutional DenseNet
SCSE: Spatial and channel squeezes and excitation
MPA: Mean pixel-wise accuracy
DSC: Dice similarity coefficient
PST: Phase stretch transform
SVM: Support vector machine
CNNs: Convolutional neural networks
FCNs: Fully convolutional networks
SW-CEL: Spatially weighted cross-entropy loss
CEL: Cross entropy loss
W-CEL: Weighted cross-entropy loss
PACS: Picture archiving and communications system
HD: Hausdorff distance
PA: Pixel-wise accuracy
GFLOPS: Giga floating-point operations per second
NPV: Negative predictive value
PPV: Positive predictive value
